# Possible requirement of executive functions for high performance in soccer

**DOI:** 10.1371/journal.pone.0201871

**Published:** 2018-08-22

**Authors:** Shota Sakamoto, Haruki Takeuchi, Naoki Ihara, Bao Ligao, Kazuhiro Suzukawa

**Affiliations:** 1 Graduate School of Health and Sport Science, Nippon Sport Science University, Setagaya-ku, Tokyo, Japan; 2 Laboratory of Chemical Pharmacology, Graduate School of Pharmaceutical Sciences, The University of Tokyo, Bunkyo-ku, Tokyo, Japan; Charite Universitatsmedizin Berlin, GERMANY

## Abstract

In open-skill sports such as soccer, the environment surrounding players is rapidly changing. Therefore, players are required to process a large amount of external information and take appropriate actions in a very short period. Executive functions (EFs)—the cognitive control processes that regulate thoughts and action—are needed for high performance in soccer. In this study, we measured the EFs of young soccer players aged 8–11 years, who were applying for admission to an elite youth program of a Japanese Football League club. We found that even though admission was determined by the soccer performance of the players, significant differences were observed between players who were approved and those who were not approved into the program. Soccer players who had been approved into the program got higher scores in general EFs tests than those who had been rejected. Our results proposed that measuring EFs provides coaches with another objective way to assess the performance levels of soccer players.

## Introduction

There is a large amount of evidence showing that sports are beneficial for both physical and mental health. In general, sports can be classified into two types: open- and closed-skill sports [1]. Open-skill sports are defined as those with a changing, unpredictable, and externally paced environment (e.g., soccer, tennis, rugby), while closed-skill sports are defined as those in which the sporting environment is relatively consistent, predictable, and self-paced (e.g., gymnastics, swimming). Among the various sports played throughout the world, soccer is clearly one of the most popular. According to a large-scale survey done by FIFA in 2006, more than 265 million people, (about 4% of the world’s population) play soccer [2].

How then can we describe “a good soccer player” in terms of the science of sports performance? There is little doubt that Cristiano Ronaldo and Lionel Messi are two of the world’s most celebrated soccer players. Their career statistics show their great talent. However, there are other players who are also considered to be great. Although there were several studies that attempted to develop a performance rating system in soccer [3–6], it is still difficult to measure soccer performance levels objectively.

It has long been thought that high performance in soccer depends on sports-specific characteristics (i.e., techniques and tactics) and physical abilities (i.e., anthropometrical, physiological, and psychological performance characteristics). In support of this idea, prior studies show that elite soccer players outperformed non-elite players in sprint performance, anaerobic capacity, and interval endurance capacity [3,7,8]. Therefore, in the past decades, players and coaches have exerted considerable effort to develop physical, technical, and tactical skills to be professionals. Recently, however, growing evidence suggests that these factors cannot fully account for success in soccer [9]. In open-skill sports such as soccer, players are required to make decisions quickly and accurately using optimum skills in a dynamically changing environment. Therefore, performance criteria are complex because many skills are influenced by overall team performance and situations. Since player decisions rely heavily on constantly changing situations, players must be able to process and recognize situations (i.e., working memory) and find out the most effective play among all conceivable choices within a short period (i.e., planning, reasoning and creativity) [10]. Furthermore, players sometimes cancel intended actions (i.e., inhibitory control) and make new decisions (i.e., cognitive flexibility and problem solving) according to rapidly changing situations. Thus, soccer players need to develop the ability to “read the game”, as well as have physical abilities.

In the field of neuropsychology, these cognitive abilities are referred to as executive functions (EFs). EFs are a set of cognitive processes that are necessary for the cognitive control of behavior [11–14]. EFs can be divided into Core EFs and Higher-order EFs [12,14]. Core EFs are comprised of three sub-functions: working memory, cognitive flexibility (also called set shifting, mental flexibility, or mental set shifting, closely linked to creativity), and inhibitory control (cognitive inhibition, self-control, and selective or focused attention). Higher-order EFs are comprised of reasoning, problem solving, and planning, which are built based on the Core EFs [11,13].

Recent studies suggest that EFs are critical for high performance in soccer. It has been shown that elite youth soccer players have better inhibitory control, cognitive flexibility, and Higher-order EFs than their sub-elite counterparts [10]. Furthermore, Vestberg’s study shows a significant correlation between results of the EFs test and the number of goals and assists the players scored two seasons later [15]. However, most of these previous studies compared EFs among pre-classified groups (e.g., elite and sub-elite athletes, high and low division players or athletes and sedentary people). In this study, we conducted general EFs tests on young soccer players aged 8–11 years old applying for admission to an elite youth program of a Japanese Football League club. After the admission decision, we compared the EFs test scores of those soccer players who had been approved to join the program and those rejected. The subject’s soccer careers vary compared to those who play together in a team. We could conduct the tests at the same time and in the same place. These proved quite beneficial in reducing sampling biases. Diamond argues that EFs are affected by social, emotional, and physical health [12,14], we assessed the mental health of the subjects using the Profile of Mood States (POMS) and the multidimensional scale of perceived social support (MSPSS) [16,17]. MSPSS is a research tool designed to assess perceptions of support from family, friends, and others. POMS is a psychological rating scale designed to measure the transient moods of the subjects. We compared their scales with EFs scores. Furthermore, Grit and Resilience were also compared between the approved and rejected groups. Grit is perseverance and passion for long-term goals and Resilience is the ability to bounce back or recover from stress, which are important mindsets for future success [18,19].

## Methods

### Ethics statement

All subjects and their parents were informed of the research procedure prior to participation. All subjects received verbal and written information about the study before giving their verbal and written informed consent to participate. This study was approved by the Ethical Committee of Nippon Sports Science University.

### Subjects

The total number of players who participated for admission into the club was 497. We excluded the players aged 6 and 7 years old because their linguistic skills were not developed enough to understand the contents of the EFs tests and questionnaires. As such, 383 male young soccer players aged 8 to 11 years old (mean age = 9.7) participated in this study. They had played soccer for several years on their respective teams. Table 1 shows the subjects’ demographic characteristics.

**Table 1:** Characteristics of subjects divided into rejected and approved groups.

	all (n = 383)	rejected (n = 187)	approved (n = 196)
	mean ± SD	mean ± SD	mean ± SD
Age (years)	9.7 ± 1.1	9.6 ± 1.1	9.8 ± 1.1
Weight (kg)	30.8 ± 5.4	31.2 ± 5.5	30.4 ± 5.2
Height (cm)	136.9 ± 8.6	137.2 ± 8.8	136.1 ± 8.4
Soccer experience (years)	4.53 ± 1.2	4.4 ± 1.2	4.6 ± 1.3

### Assessment of soccer performance

Eight professional coaches with more than 10 years of high-level coaching experience and official license from the Japanese Football Association (JFA) assessed soccer performance levels of the subjects. These coaches observed the subjects playing soccer (50 meters × 30 meters field size, 7 vs. 7 without goalkeepers) to assess their soccer performance levels. All games were recorded with iPads. To reduce biased viewpoints, the subjects were assessed by a consensus of opinions from the eight coaches. It should be noted that EFs tests, and questionnaires were not considered for admission. All subjects played in 10-15-minute games three times.

### Assessment of Core and Higher-order EFs

#### Core EFs

To evaluate Core EFs, we adopted the Stroop test. The Stroop test measures inhibitory control and information processing speed/accuracy [20–22]. Our Stroop test was non-verbal test in which subjects used their hands and a pen. This test requires manually selecting a color patch corresponding to a color-word combination’s semantic meaning or selecting a word patch corresponding to the ink color of the color-word combination. It consists of four tasks. Five colors (red, yellow, blue, green and black) were used as the five stimuli in the task. In task 1 (control task) and task 2 (incongruent task), the subjects checked the color patch corresponding to the color word from among the five choices of color patches. In task 1, the color words were written with black ink. Task 2, words were written with incongruent color ink (e.g., the word “red” was printed in yellow). Task 3 (control task) and task 4 (incongruent task), the subjects checked the color word corresponding to the color of ink in the leftmost patch from among the five choices of color words that were written in black ink on the right-hand side. Task 4 (incongruent task) followed the same instructions as task 3, however, the color words were written with incongruent color ink (e.g., the word “red” was printed in blue). The subjects were required to check the color or color word that corresponded to the stimuli as quickly and as accurately as possible within 60 sec. All color-word combinations and color patches were printed on four separate sheets of paper. Each test consists of 10 practice trials and 100 test trials. The subjects were required to select the color patch as fast as possible. We compared the number of correct responses across the four conditions between the rejected and approved groups. The subjects with correct answer rate below chance level (<20%) were excluded from the analysis.

#### Higher-order EFs

We adopted the Design Fluency Test (DFT) to measure Higher-order EFs [23–25]. DFT is known to assess planning, cognitive flexibility, creativity, and working memory. Subjects draw as many different designs as possible in a minute, while avoiding repeating prior designs. DFT is a non-verbal psychomotor test in which subjects use their hands and a pen to connect dots using lines. Five dots were arranged in a square frame. The task was to find as many different combinations of connecting the dots with four lines as possible under time pressure (60 sec). The subjects were not allowed to repeat any figures. Therefore, the DFT required the subjects to remember previous figures and create new ones (i.e. not repeat the previous combinations). The total number of correct and incorrect figures was counted.

### Questionnaire

The questionnaires were formatted by online questionnaire software, SurveyMonkey. The questionnaires were composed of closed questions. The subjects were asked to answer the questions for Grit, MSPSS, Resilience and POMS.

#### Grit

Grit is defined as perseverance and passion for long-term goals [18]. Several studies suggest that Grit is required for success in life [26,27]. The Grit scale consisted of 12 items: 1) I often set a goal but later choose to pursue a different one, 2) New ideas and projects sometimes distract me from previous ones, 3) I become interested in new pursuits every few months, 4) My interests change from year to year, 5). I have been obsessed with a certain idea or project for a short time but later lost interest, 6) I have difficulty maintaining my focus on projects that take more than a few months to complete, 7) I have achieved a goal that took years of work, 8) I have overcome setbacks to conquer an important challenge, 9) I finish whatever I begin, 10) Setbacks don’t discourage me, 11) I am a hard worker, and 12) I am diligent [28]. This questionnaire was composed of two factors. The first one consists of 6 items indicating consistency of “Interests,” and the other one also consists of 6 items indicating perseverance of “Effort”. To test the integrity of the final two-factor solution, we confirmed that the specificity of each factor. Each factor had a range of points among 1–5. Each point described a level of Grit as follows: 1 (not like me at all) to 5 (very much like me). The Grit scores were calculated as the average of all 12 items, with higher values representing higher levels of Grit. (Cronbach’s alpha = 0.78).

#### The multidimensional scale of perceived social support (MSPSS)

The MSPSS in Japanese [17] was used to evaluate social support. The MSPSS consists of 12 items: 1) There is a special person who is around when I am in need, 2) There is a special person with whom I can share my joys and sorrows, 3) My family really ties to help me, 4) I get the emotional help and support I need from my family, 5) I have a special person who is a real source of comfort to me, 6) My friends really try to help me, 7) I can count on my friends when things go wrong, 8) I can talk about my problems with my family, 9) I have friends with whom I can share my joys and sorrows, 10) There is a special person in my life who cares about my feelings, 11) My family is willing to help me make decisions, and 12) I can talk about my problems with my friends. This scale measured perceived social support from family, friends, and significant others. Subjects used a 7-point scale (1: very strongly disagree to 7: very strongly agree) for each item. (Cronbach’s alpha = 0.87).

#### Resilience

Resilience is defined as the ability to successfully cope with change or misfortune. Resilience of the subjects was evaluated by a questionnaire [19,29]. The Resilience Scale had 21 items classified into three subscales of Novelty Seeking, Emotional Regulation, and Positive Future Orientation. The items are: 1) I seek new challenges, 2) I think I can control my emotions, 3) I am sure that good things will happen in the future, 4) I like new or intriguing things, 5) I can stay calm in tough circumstances, 6) I think I have a bright future, 7) I think I have a high level of interest and curiosity, 8) I make an effort to always stay calm, 9) I feel positive about my future, 10) I like to find out about things, 11) I think I have perseverance, 12) I have a clear goal for the future, 13) I think difficulties form a part of life’s valuable experiences, 14) I find it difficult not to dwell on negative experience, 15) I am striving towards my future goal, 16) I don’t like to do unfamiliar things, 17) I cannot endure adversity, 18) I find it bothersome to start new activities, 19) My behavior varies with my daily moods, 20) I lose interest quickly, and 21) I have difficulty in controlling my anger. Each item described a level of resilience on a scale of 1 (Definitely no) to 5 (Definitely yes) (Cronbach’s alpha = 0.84).

#### POMS

The Profile of Mood States (POMS) is a sensitive measure of mood in normal healthy subjects [16]. This measure consisted of 6 subscales: tension-anxiety (e.g., nervous, anxious), depression-dejection (e.g., sad, unworthy), anger-hostility (e.g., grouchy, furious), vigor-activity (e.g., lively, active), fatigue inertia (e.g., tired, sluggish), and confusion-bewilderment (e.g., muddled, forgetful). The score of POMS can be used as the index of allover stress reaction. Each item described a level of mood as follows: 0 (Definitely no) to 4 (Definitely yes) (Cronbach’s alpha = 0.77).

### EFs composite score

To measure general EFs level of the subjects, we introduced the EFs composite score, which was obtained by adding the standard score (z-score) of the number of correct answers in the Stroop test and the DFT [30]. To obtain z-scores of the Stroop test, we took the average of correct answers of task 2 and 4.

### Procedure

The EFs tests (Stroop test and DFT) and Questionnaires (Grit, MSPSS, Resilience, and POMS) were administered in a quiet room before the soccer games. Admission to the club was decided by the soccer performance of the subjects during games, and the coaches did not provide training or coaching to any players. The two EFs tests (Stroop test and DFT) and questionnaires were not considered for admission to the club. The admission results were announced several weeks after completion of the tests. The selection tests were held on February 14, 15, 17, and 19, 2017.

### Statistical analysis

Missing values were excluded from analyses. Cohen’s *d* effect sizes of each test were calculated. Two-tailed *t*-tests were performed to determine statistical significance. The Pearson correlation coefficient was calculated between the EFs composite scores and the scales of Grit, MSPSS, Resilience, and POMS. Cronbach’s alphas were calculated to confirm the reliability of the scales. Significance levels were set at p < 0.05. All statistical analyses were conducted using OriginPro 2016 and SPSS version 25.

## Results

Based on soccer performance levels, the subjects applying for admission to an elite youth program were divided into two groups: rejected and approved. Several variables were compared between these two groups, and significant differences were observed in the EFs tests, and resilience (Table 2).

**Table 2:** Summary of descriptive statistics for all test scores in each group.

		rejected	approved	Statistics	Cohen's *d*
		mean ± SD	mean ± SD		
Stroop test	task 1	40.9 ± 10.3	42.9 ± 7.3	t(254) = 1.85; p = 0.065	0.23
	task 2	31.3 ± 9.6	34.5 ± 8.6	t(352) = 3.24; **p = 0.001	0.35
	task 3	33.8 ± 6.5	34.6 ± 5.9	t(253) = 1.10; p = 0.269	0.14
	task 4	26.0 ±7.3	28.2 ± 7.4	t(346) = 2.75; **p = 0.006	0.30
DFT	correct	6.79 ± 3.04	7.72 ± 3.03	t(379) = 3.00; **p = 0.003	0.31
	incorrect	1.49 ± 2.36	1.45 ± 2.48	t(379) = -0.17; p = 0.858	0.143
GRIT		3.92 ± 0.45	3.82 ± 0.45	t(312) = -1.96; p = 0.051	-0.221
Social support		6.36 ± 0.60	6.33 ± 0.52	t(312) = -0.46; p = 0.644	-0.052
Resilience		3.47 ± 0.32	3.38 ± 0.34	t(312) = -2.44; *p = 0.015	-0.275
POMS		-3.33 ± 10.42	-2.37 ± 8.63	t(312) = 0.89; p = 0.376	0.100

DFT = Design Fluency Test. POMS = Profile of Mood States.

* p < 0.05.

** p < 0.01.

### Stroop test

**Fig 1:**
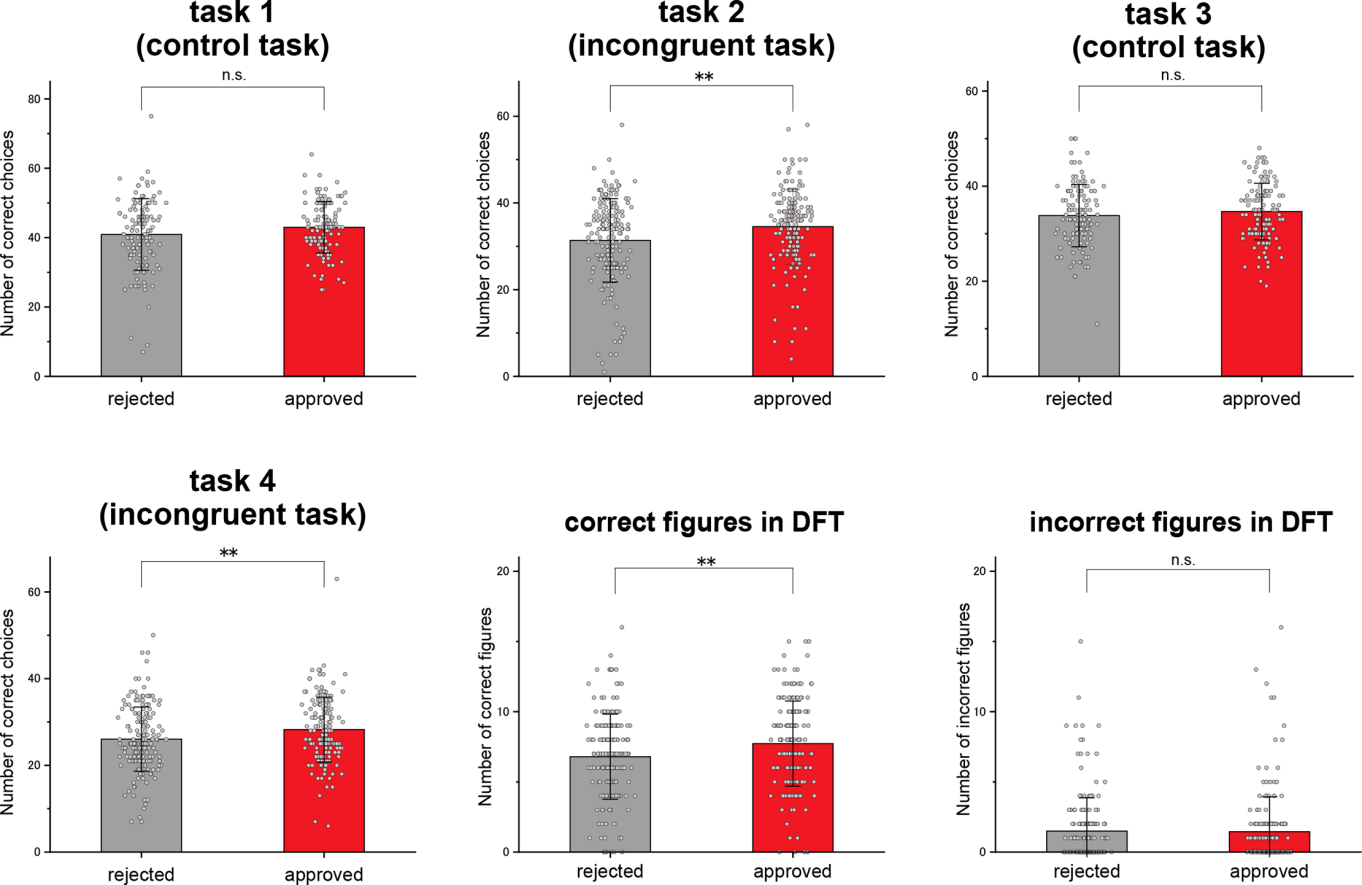
Executive functions in the rejected and approved groups. Significant differences were observed in the Stroop test (task 2 and 4), and DFT. Error bars indicate SD. DFT = Design Fluency Test. n.s., not significant. ** p < 0.01.

To measure the processing speed/accuracy and inhibitory control, we counted the total number of correct choices. No significant differences were observed between the rejected and the approved groups in task 1 and 3 (control tasks). However, there were significant differences in task 2 and 4 (incongruent tasks). The approved group showed better scores in the both tests than the rejected group (Fig 1 and Table 2).

### Design Fluency Test (DFT)

The total number of correct and incorrect figures was counted and compared between the rejected and the approved groups. A significant difference was observed in the number of correct figures but not incorrect ones. The approved group showed better scores than the rejected group in the test (Fig 1 and Table 2).

### Questionnaire

To evaluate mental health of the subjects, we asked them to complete the questionnaires (i.e., Grit, MSPSS, Resilience and POMS). No difference between the approved and rejected groups was found for Grit, MSPSS, and POMS. However, the rejected group showed better scores in Resilience than the approved group (S1 Fig and Table 2).

### Relationship between EFs level and mental health

To measure general EFs level, we calculated the EFs composite score, which was obtain by adding the standard score (z-score) of the number of correct answers in the Stroop test and the DFT [30]. We then compared the EFs composite score with the score of Grit, MSPSS, Resilience, and POMS. No significant correlation was found between them (Grit: r = -0.08, MSPSS: r = 0.01, Resilience: r = 0.03, POMS: r = -0.04) (S2 Fig).

## Discussion

In this study, we measured the EFs of young soccer players applying for admission to an elite youth program of a Japanese Football League club. Even though the admission was determined by subjects’ soccer performance during games, the approved group significantly outperformed the rejected group in the Stroop Test and the DFT. This result was consistent with previous studies that demonstrated elite young soccer players showed higher scores in general EFs tests than non-elite youth players [10,30,31]. Similar results have also been reported in other sports such as hockey and rugby [32,33]. These results strongly suggest that EFs are necessary for high performance in open-skill sports. Most of previous studies compared EFs among pre-classified groups (e.g., elite and sub-elite athletes, high and low division players or athletes and sedentary people) [10,30,31]. To reduce pre-existing environmental biases, we conducted the EFs test for junior soccer players who applied for admission into the club and examined whether these tests could be used as one of the admission tests. The results showed that the approved group got better scores than the rejected group in the tests. The differences were statistically significant but effect sizes were small. We could not predict the acceptance or rejection of the soccer players just based on the EFs test scores. However, the EFs tests can measure a different aspect of the brain function, which cannot be assessed by the commonly-prescribed physical tests. In addition, these tests can be easily carried out to large number of soccer players with paper and pencil. We would like to propose that measuring EFs provides coaches with another objective way to assess the performance levels of soccer players.

The Stroop Test mainly measures the processing speed and inhibitory control, which belong to Core EFs [20–22]. The DFT captures cognitive flexibility, focus, sustained attention and working memory [23,24]. Rather, the main measurement of the DFT is creativity because the brain needs to manipulate data and change the action constantly in line with the incoming data mixed with old data to find new solutions [25]. Therefore, the DFT measures Higher-order EFs. In the DFT, we also counted the total number of incorrect figures in addition to correct figures. Since most incorrect figures were made by repeating a design, we assumed that incorrect figures could be attributed to poor working memory or weaker focus in the visual attention. However, no significant difference was observed in the number of incorrect figures between the rejected and approved groups (Fig 1 and Table 2). This result suggests that the level of working memory and focus were comparable between these two groups. It is likely that the level of creativity and cognitive flexibility, rather than working memory or focus, are higher in the approved group than in the rejected group. At any rate, we found significant differences between the approved and the rejected groups in the Stroop test and DFT. These findings suggest that both Core EFs and Higher-order EFs coordinately have effects on soccer performance.

Since EFs are affected by social, emotional, and physical health are important for cognitive health because stress, loneliness, and lack of exercise or sleep impair EFs [12,14]. Therefore, we performed MSPSS and POMS to monitor subjects’ mental health. MSPSS is a research tool designed to assess perceptions of support from family, friends, and others. POMS is a psychological rating scale designed to measure the transient moods of the subjects. No difference was found in the stress level and social support between the rejected and approved group, suggesting that the differences between them in the EFs tests can be attributed to EFs levels. Furthermore, we could not find any correlation between the psychological questionnaires scales and the EFs tests scores (2 Fig). The differences observed in the current psychological questionnaires among the subjects are not likely to be significant enough to affect EFs. We also measured Grit and Resilience, both of which are known to be important for future success [26,27,19]. Grit is defined as perseverance and passion for long-term goals [18], and Resilience is referred to as the emotional strength that allows an individual to recover quickly from change, misfortune, and bounce back from adversity [19]. Based on these previous studies [26,19], we initially expected that the approved group showed better scores in both tests. However, the rejected group showed better scores in Resilience than the approved group (S1 Fig and Table 2). Similar tendency was also observed in Grit (S1 Fig and Table 2). The psychosocial factors associated with resilience and Grit include optimism and psychological well-being. It is possible that pessimistic ways of thinking make players unsatisfied with the current situation and work harder to improve themselves.

We should acknowledge several limitations of our study. First, it was difficult to assess soccer performance in a way other than subjectively because performance levels were judged by coaches. To reduce biased viewpoints, we assessed the subjects using a consensus of opinions from eight professional coaches. A More objective method has been employed, and includes counting the number a player’s goals and assists to assess soccer performance levels [15,30]. However, players’ roles vary during games, so evaluating a player’s performance solely by counting the number of their plays involved in goals is not sufficient. More factors should be considered for describing soccer performance characteristics. Second, our present findings cannot mention the predictive ability of EFs for future success in soccer, because EFs develop progressively from childhood through adolescence [34]. Furthermore, maturation speed of EFs might vary among individuals. Thus, even if higher levels of EFs are necessary for good soccer performance, we cannot predict how many players from the approved players will be successful as professional soccer players in the future. Third, we conducted a cross-sectional research study. Therefore, we could not address the causality between EFs and soccer performance level. It is possible that the EFs plays critical roles in high soccer performance, or that engaging in soccer improves the EFs. It remains controversial whether EFs are trainable. Several studies with twins indicate that EFs are mainly pre-determined by genetic factors [35,36]. Nonetheless, there is some evidence that physical exercise improves EFs [37–39]. It would be very interesting to examine whether engaging in soccer can improve general EFs because EFs levels also correlate with success in a daily life. At any rate, longitudinal and intervention studies are necessary to address these possibilities.

## Supporting information

S1 Fig Questionnaire scores in the rejected and the approved groups. There was significant difference in Resilience. * p < 0.05.
(TIF)

S2 Fig Relationship between EFs level and mental health. The Pearson correlation coefficient was calculated between the EFs composite scores and the scales of Grit, MSPSS, Resilience, and POMS.
(TIF)

S1 File Dataset. (XLSX)
