# Correction: Possible requirement of executive functions for high performance in soccer

**DOI:** 10.1371/journal.pone.0251168

**Published:** 2021-04-29

**Authors:** Shota Sakamoto, Haruki Takeuchi, Naoki Ihara, Bao Ligao, Kazuhiro Suzukawa

Reference 29 in the published article [1] is incorrect. The reference should instead link to the updated version of the Adolescent Resilience Scale, which is available at http://www.f.waseda.jp/oshio.at/research/scales/ARS_in_English.pdf [2]. The authors of the study used the updated version of the scale in their study.
