# Supplementary figures and images for: Possible requirement of executive functions for high performance in soccer
Source: PLoS One. 2018 Aug 22;13(8):e0201871. doi: 10.1371/journal.pone.0201871 (PMC6104941; doi:10.1371/journal.pone.0201871)

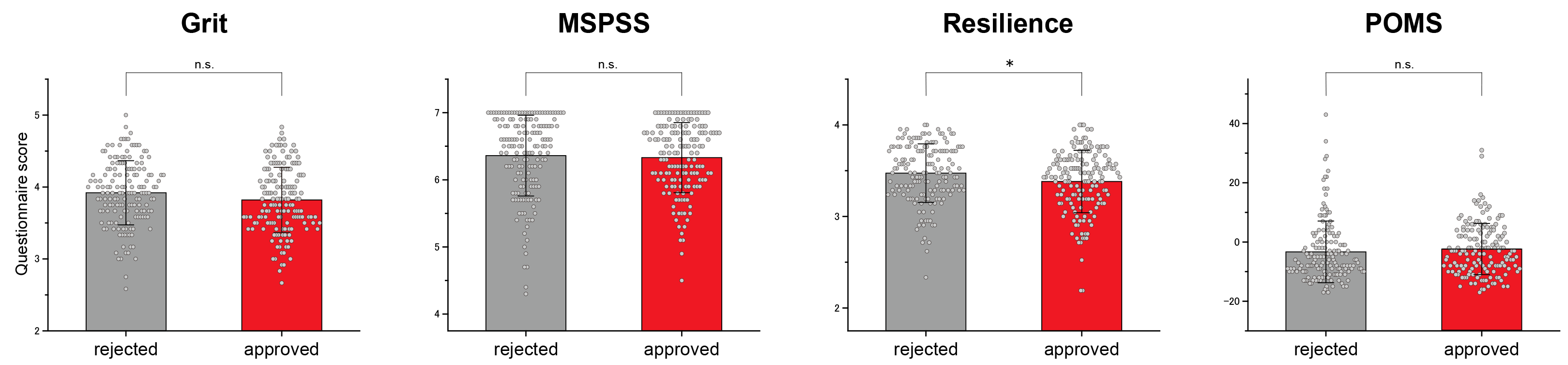

Supplement: S1 Fig — There was significant difference in Resilience. * p < 0.05. (TIF) [file pone.0201871.s001.tif]

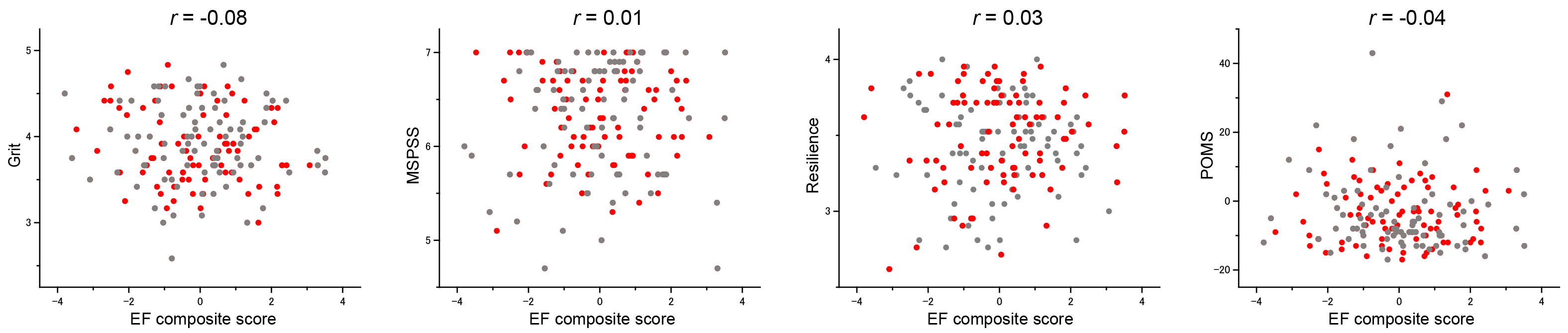

Supplement: S2 Fig — The Pearson correlation coefficient was calculated between the EFs composite scores and the scales of Grit, MSPSS, Resilience, and POMS. (TIF) [file pone.0201871.s002.tif]
